# Astragaloside IV ameliorates allergic inflammation by inhibiting key initiating factors in the initial stage of sensitization

**DOI:** 10.1038/srep38241

**Published:** 2016-12-05

**Authors:** Kai-fan Bao, Xi Yu, Xiao Wei, Li-li Gui, Hai-liang Liu, Xiao-yu Wang, Yu Tao, Guo-rong Jiang, Min Hong

**Affiliations:** 1 Jiangsu Key Laboratory of Pediatric Respiratory Disease, Jiangsu Key Laboratory for Pharmacology and Safety Evaluation of Chinese Materia Medica, Nanjing University of Chinese Medicine, Nanjing, 210046, China; 2 Suzhou Hospital of Traditional Chinese Medicine, Suzhou, 215009, China

## Abstract

To illuminate the anti-allergy mechanism of astragaloside IV (AS-IV), we assessed its effects in a murine model of allergic contact dermatitis (ACD). AS-IV administered in the sensitization phase, rather than in the elicitation phase, dramatically alleviated the symptoms of allergic inflammation. We hypothesized that AS-IV exerts its anti-allergy effects by regulating the production of key pro-allergic cytokines based on the fact that interleukin (IL)-33 and thymic stromal lymphopoietin (TSLP) levels increase significantly in the initial stage of the sensitization phase. AS-IV administered in the initial stage of ACD inhibited TSLP and IL-33 expression and reduced the proportion of type-2 innate lymphoid cells (ILC2s). An *in vitro* study showed that the production of pro-allergic cytokines was significantly inhibited in AS-IV presenting HaCaT cells. We also verified that AS-IV administered only in the initial stage markedly alleviated inflammation, including ear swelling, Th2 cytokine expression, and histological changes. Taken together, these results suggest that AS-IV effectively ameliorates the progression of allergic inflammation by inhibiting key initiating factors, including TSLP and IL-33, and can be used to prevent and/or treat patients with ACD. Our data also suggest that these key pro-allergic cytokines are potential therapeutic targets for allergic diseases.

Allergic diseases are a significant health problem today in both developed and developing countries^1^. Allergic diseases are highly patient specific and include asthma, food allergies, and atopic dermatitis (AD)^2^. They have reached epidemic proportions worldwide and their incidence is continuing to increase in association with modern lifestyles^3^. The mechanism underlying the recurrence of allergic diseases is far from resolved^4^,^5^,^6^, making it urgent to explore the mechanisms of allergic diseases and to develop new drugs that effectively reduce the rate of recurrence.

*Astragalus membranaceus*, also called ‘Huangqi’ in Chinese, is commonly used in Chinese medicinal formulae for the treatment of several allergic diseases, including asthma, Henoch–Schonlein purpura nephritis, rhinitis^7^,^8^,^9^. Astragaloside IV (AS-IV) is a saponin purified from Huangqi, and is regarded as the quality standard for *A. membranaceus* injection in the Pharmacopeia of the People’s Republic of China. Previous studies have shown that AS-IV exerts a potent anti-allergic effect on ovalbumin-induced asthma^10^ and seasonal allergic rhinitis^9^. Because Huangqi reduces the recurrence of allergic diseases, we hypothesized that AS-IV, the main active component in the herb, plays an important role in regulating allergic diseases in their initial stages, before the acute phase.

Barrier epithelial cells (ECs) represent the very first line of defense and express pattern recognition receptors that recognize type-2-cell-mediated immune insults, such as allergens^11^. Interleukin 33 (IL-33), a cytokine of the IL-1 family, is mainly derived from epithelial cells. It is an alarmin cytokine that plays a crucial role in the initiation of type-2 immune responses following infection with parasites or viruses or exposure to allergens^12^. Interleukin (IL)-33 is a key cytokine in the induction and activation of type-2 innate lymphoid cells (ILC2s), which produce large amounts of type-2 cytokines, such as IL-5 and IL-13, in allergic disorders^13^. IL-33 is a potent stimulator of skin ILC2s, and the absence of IL-33 signaling reduced skin inflammation in a mouse model of AD^14^. Similarly, the levels of IL-33 were higher in the lung smooth muscle cells and ECs of asthmatic patients than in those of healthy subjects^15^,^16^,^17^.

Thymic stromal lymphopoietin (TSLP) is another crucial epithelium-derived cytokine. It exerts its biological functions by binding to the TSLP receptor (TSLPR) and activates dendritic cells (DCs) to induce naïve CD4^+^ T cells to differentiate into inflammatory Th2 cells by upregulating the expression of the tumor necrosis factor (TNF) superfamily protein OX40L^18^,^19^,^20^. Accumulating evidence indicates that TSLP is crucial for allergic diseases such as AD, asthma, and allergic rhinoconjunctivitis^21^. As a specific promoter of allergic inflammation, TSLP often acts in a positive feedback loop, amplifying and leading to the chronicity of Th2 inflammatory responses^11^,^22^. TSLP is highly expressed in the keratinocytes of allergic contact dermatitis (ACD) lesions and its expression correlates with the severity of ACD, not only in animal models, but also in patients^23^,^24^. Furthermore, TSLP acts as a potent stimulator of Th2 cytokines and induces the proliferation, differentiation, and activation of mast cells^25^. Thus, TSLP may act as a master switch that triggers the initiation and maintenance of ACD.

We hypothesized that AS-IV could regulate the key pro-allergic cytokines IL-33 and TSLP derived from ECs and as a consequence, inhibits the occurrence and development of allergic diseases. Fluorescein isothiocyanate (FITC)-induced ACD, which is a Th2-dominant disease^26^, was selected as a typical model of allergic disease. In contrast to 2,4-dinitrochlorobenzene (DNCB)-induced Th1-dominant ACD, the FITC-induced ACD model appropriately reproduces the key features of allergic inflammation. In this study, we tested the effects of AS-IV after different periods of exposure on various phases of the FITC-induced ACD model and investigated the underlying mechanisms.

## Results

### AS-IV administered during the whole course of ACD inhibits associated inflammation

**Figure 1:**
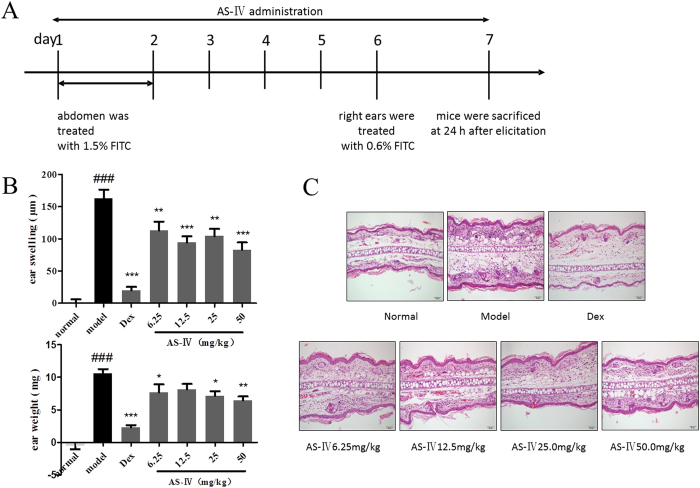
AS-IV administered during the whole experiment inhibited ACD-associated inflammation. (**A**) Flow charts of the FITC-induced ACD model and AS-IV was administered during the 7 days of the experiment. (**B**) Ear swelling and ear weight were measured on day 7 in the ACD model mice (means + SD, n = 10, ^###^P < 0.001 *vs* normal; *P < 0.05, **P < 0.01, ***P < 0.001 *vs* model) (**C**) Hematoxylin and eosin (H&E)-stained ear skin sections from FITC-induced ACD model mice (n = 5; magnification: ×200).

To examine the effect of AS-IV on ACD, mice were treated daily with AS-IV for 7 days during the course of the experiment. Ear tissues were collected 24 h after the final FITC challenge (Fig. 1A). We tested the morphological changes and determined the cytokine levels in the mouse ear homogenates. The suppression of inflammation by AS-IV in mice with FITC-induced ACD was estimated by measuring ear swelling and ear weight (Fig. 1B). A histological analysis indicated that AS-IV effectively alleviated FITC-induced inflammatory cell infiltration and edema (Fig. 1C).

### AS-IV given in the sensitization phase, rather than the elicitation phase, markedly alleviated inflammation

To determine the phase of the allergic response in which AS-IV significantly alleviates inflammation, we compared a series of indices of allergic inflammation in the sensitization and elicitation phases.

**Figure 2:**
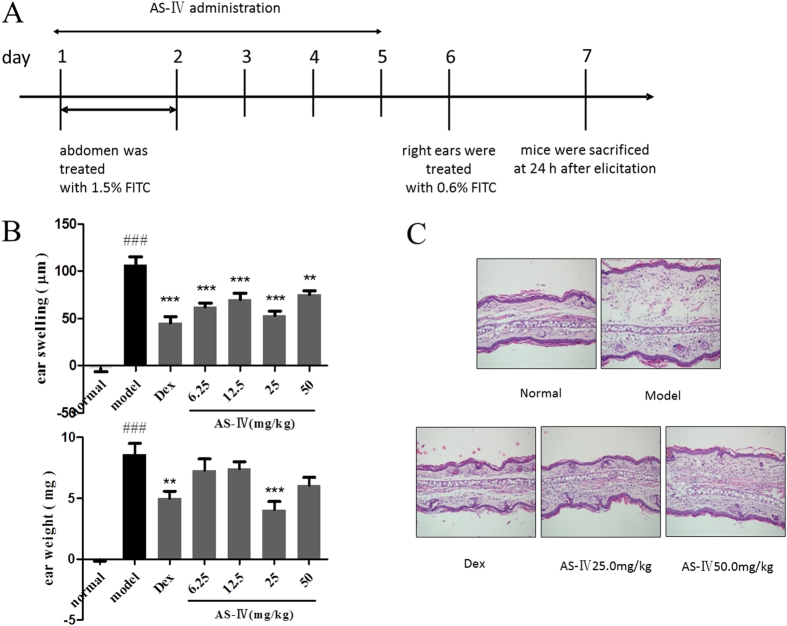
AS-IV administered in the sensitization phase inhibited FITC-induced ACD. (**A**) Flow charts of the FITC-induced ACD model and AS-IV was administered in the sensitization phase. Mice were topically sensitized with 1.5% FITC solution applied to the abdominal skin on days 1 and 2. AS-IV (6.25, 12.5, 25, or 50 mg/kg, intragastrically) was administered from day 1 to day 5. The mice were killed on day 7. (**B**) Effects of AS-IV on ear swelling and ear weight in the ACD model (mean + SD, n = 10, ^###^P < 0.001 *vs* normal; **P < 0.01, ***P < 0.001 *vs* model). (**C**) Effect of AS-IV on the histopathology of H&E-stained ear skin sections (n = 5; magnification: ×200).

As shown in Fig. 2A, we modulated the dose regimen and observed the effects of AS-IV administered in the sensitization phase on ear inflammation by measuring ear swelling and ear weight. The administration of 25 mg/kg AS-IV in the sensitization phase significantly inhibited the ear inflammation induced by FITC (Fig. 2B). A histological examination showed that 25 mg/kg AS-IV at markedly alleviated ear swelling and the infiltration of inflammatory cells to levels similar to those in normal ears (Fig. 2C).

**Figure 3:**
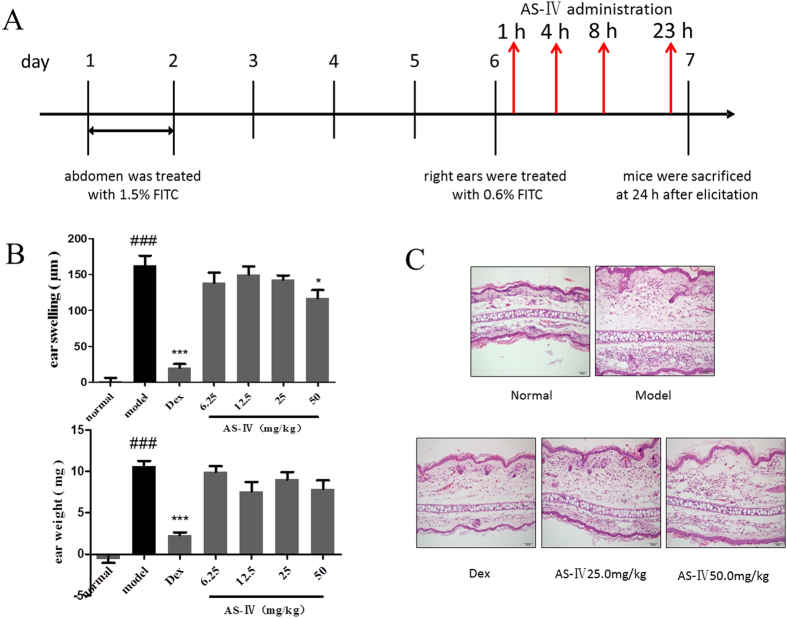
AS-IV administered in the elicitation phase did not significantly affect FITC-induced ACD. (**A**) Flow charts of the FITC-induced ACD model and AS-IV was administered four times at 1, 4, 8, and 23 h after elicitation on day 6. The mice were sacrificed on day 7. (**B**) Effects of AS-IV on ear swelling and ear weight in the ACD model (mean + SD, n = 10, ^###^P < 0.001 *vs* normal; *P < 0.05, ***P < 0.001 *vs* model). (**C**) Effect of AS-IV on the histopathology of H&E-stained ear skin sections (n = 5; magnification: ×200).

We also administered AS-IV in the elicitation phase; a dose was administered before elicitation with 0.6% FITC on day 6 and thrice thereafter (Fig. 3A). The mice were sacrificed 24 h after elicitation. As shown in Fig. 3B and C, AS-IV inhibited FITC-induced ear swelling and the infiltration of inflammatory cells to some extent, but was not as effective as in the sensitization phase.

### IL-33 and TSLP increased significantly in the initial stage of the sensitization phase of FITC-induced ACD, without significant ear inflammation

**Figure 4:**
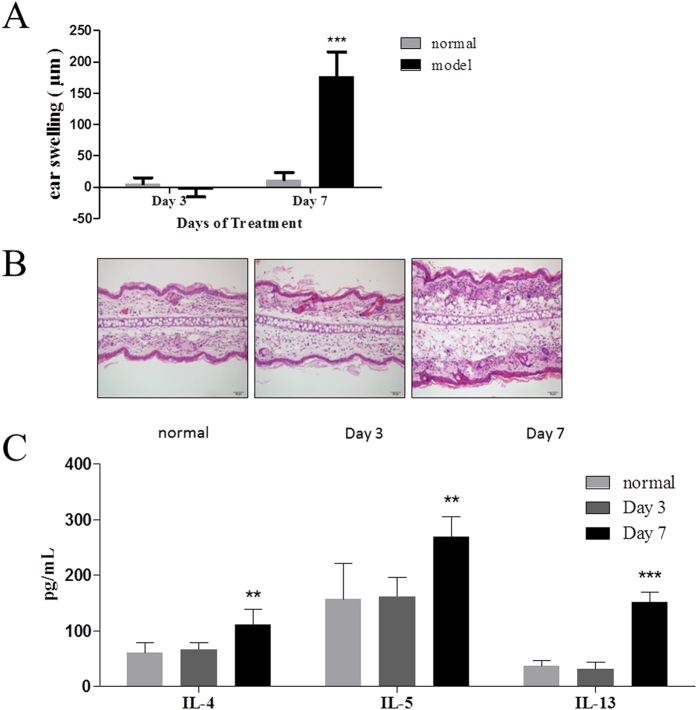
Changes in ear tissues and Th2 cytokines in different phases of the FITC-induced ACD mouse model. Day 3 represents the initial stage of the sensitization phase and day 7 represents the elicitation phase. Mice were sensitized with 1.5% FITC solution applied to the abdominal skin on days 1 and 2 and an allergic response was elicited with 0.6% FITC solution applied to the right ears on day 6. The mice were killed on day 3 or day 7. (**A**) Ear swelling in different phases of the FITC-induced ACD model. (**B**) Histopathological findings in H&E-stained ear skin sections (n = 5; magnification: ×200). (**C**) Th2 cytokine levels in different phases of the FITC-induced ACD mouse model (mean + SD, n = 10, **P < 0.01, ***P < 0.001 *vs* normal).

As we noticed that AS-IV administered in the sensitization phase significantly alleviated FITC-induced inflammation, we explored the pathogenesis of this kind of allergy. We measured ear swelling on days 3 and 7, which represent the initial stages of the sensitization phase and elicitation phase, respectively. A histological analysis of ear skin sections showed that the ears did not swell on day 3, but showed severe inflammation on day 7, when exposed locally to FITC (Fig. 4A and B). We also measured the levels of IL-4, IL-5, and IL-13 in the ear tissues in these two phases. On day 7, the Th2 cytokines were markedly increased, but were not detected in the tissues collected on day 3 (Fig. 4C). Therefore, local exposure to FITC induced the steady severe allergic inflammation that occurs in ACD. However, there were no obvious morphological changes in the initial stage of the sensitization phase.

**Figure 5:**
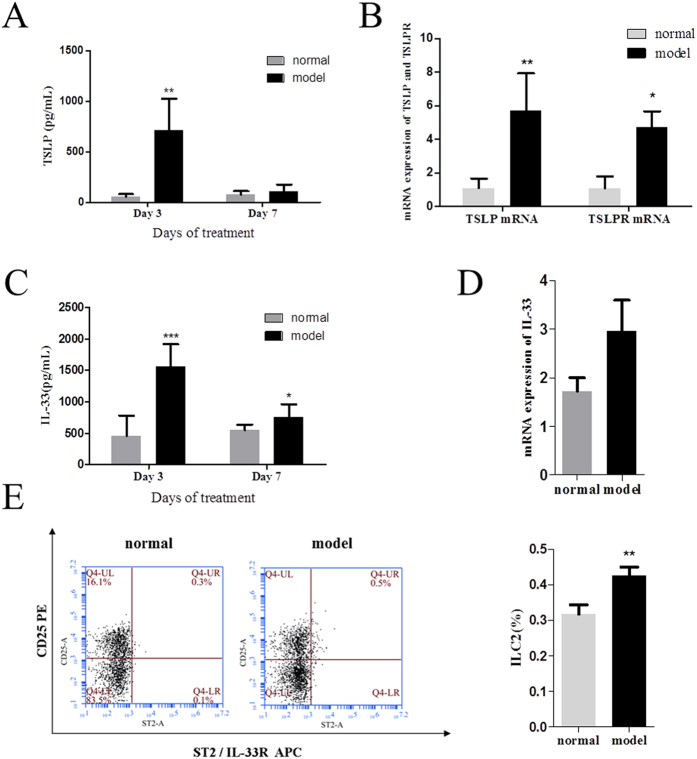
Levels of IL-33 and TSLP increased in the initial stage of the sensitization phase of FITC-induced ACD and declined in the elicitation phase. Day 3 represents the initial stage of the sensitization phase and day 7 represents the elicitation phase. (**A**) Expression of TSLP on day 3 and day 7 of the FITC-induced ACD model. (**B**) Expression of TSLP and TSLPR mRNAs on day 3. (**C**) Expression of IL-33 in different phases of the FITC-induced ACD mouse model. (**D**) Expression of IL-33 mRNA on day 3. (**E**) Changes in ILC2s (Lin^−^CD25^+^ST2^+^) in the sensitization phase (mean + SD, n = 10, *P < 0.05, **P < 0.01, ***P < 0.001 *vs* normal).

Interestingly, IL-33 and TSLP showed a different trend from that of the Th2 cytokines in the FITC-induced ACD model. As shown in Fig. 5A, TSLP was strongly expressed in the ear skin on day 3, even when the mice were only exposed to FITC on the abdomen, and decreased to almost the normal level on day 7 after local challenge. The TSLP and TSLPR mRNAs were markedly increased on day 3 (Fig. 5B). These data suggest that TSLP expression is stimulated by allergens, and that TSLP then binds to its receptor to trigger allergic inflammation. IL-33 showed a basically similar tendency. Although IL-33 remained at a level higher than in normal tissues (P < 0.05), it declined sharply compared with its expression on day 3 (Fig. 5C). The expression of IL-33 mRNA also increased to some extent (Fig. 5D). A flow-cytometric analysis showed that the proportion of ILC2s (Lin^−^CD25^+^IL-33R^+^) in the ear-skin-draining lymph nodes of the model mice was increased markedly on day 3, as shown in Fig. 5E. This suggests that with abdominal exposure to FITC, IL-33 increased systemically and then activated ILC2s. As a result, it triggers the initiation and maintenance of ACD.

### AS-IV inhibited TSLP and IL-33 expression and reduced the proportion of ILC2s in the initial stage of the sensitization phase of ACD

We demonstrated the strong expression of IL-33 in the initial stage of ACD and its decline in the elicitation phase. A similar trend in TSLP expression was also observed in the process of ACD. Therefore, we hypothesized that AS-IV inhibits ACD by regulating the expression of key pro-allergic cytokines.

**Figure 6:**
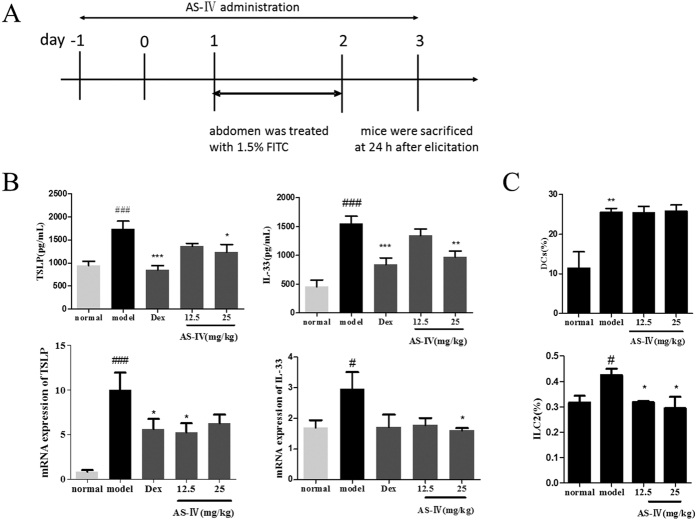
Effects of AS-IV administered in the initial stage of ACD on IL-33, TSLP, and their effector cells. Mice were topically sensitized with 1.5% FITC solution applied to the abdominal skin on days 1 and 2. Different doses of AS-IV were administered from day −1 to day 3. (**A**) Flow charts of the sensitization phase of the FITC-induced ACD model and when AS-IV was administered in the initial stage. (**B**) Effects of AS-IV on IL-33, TSLP, and their mRNA levels. (**C**) Effects of AS-IV on DCs (CD11C^+^CD40^+^CD86^+^) and ILC2s in the sensitization phase of the ACD model (mean + SD, n = 10, ^#^P < 0.05, ^##^P < 0.01, ^###^P < 0.001 *vs* normal; *P < 0.05, **P < 0.01, ***P < 0.001 *vs* model).

AS-IV was given at the initial stage of the sensitization phase (Fig. 6A), and we examined the changes in TSLP and IL-33 mRNA and protein levels in ear tissue homogenates. AS-IV, to varying degrees, reduced TSLP and IL-33 compared with their levels in the untreated model group. Similar changes in mRNA levels were observed (Fig. 6B). Therefore, AS-IV may regulate the expression of key pro-allergic cytokines in the initial stage of the sensitization phase of ACD, which is probably associated with its alleviation of ear swelling and inflammation. Furthermore, as shown in Fig. 6C, the proportions of DCs and ILC2s in the initial stage of the ACD model were markedly higher after treatment with AS-IV than in the normal group. The AS-IV-treated groups showed no significant changes in the proportion of DCs. In contrast, the proportion of ILC2s was significantly lower in the draining lymph nodes of the AS-IV-treated mice than in those of the model group. Therefore, AS-IV inhibits the production of some key pro-allergic cytokines and reduces their target cell numbers in the initial stage of the sensitization phase.

### AS-IV inhibits TSLP and IL-33 production *in vitro*

Previous studies have shown that IL-33 and TSLP are functionally involved in the pathogenesis of ACD, especially in keratinocytes^27^,^28^. Therefore, to confirm the effects of AS-IV on the expression of TSLP and IL-33, we examined the effects of AS-IV on the poly(I:C)- and TNF-α-induced production of TSLP and IL-33 in HaCaT cells *in vitro*.

**Figure 7:**
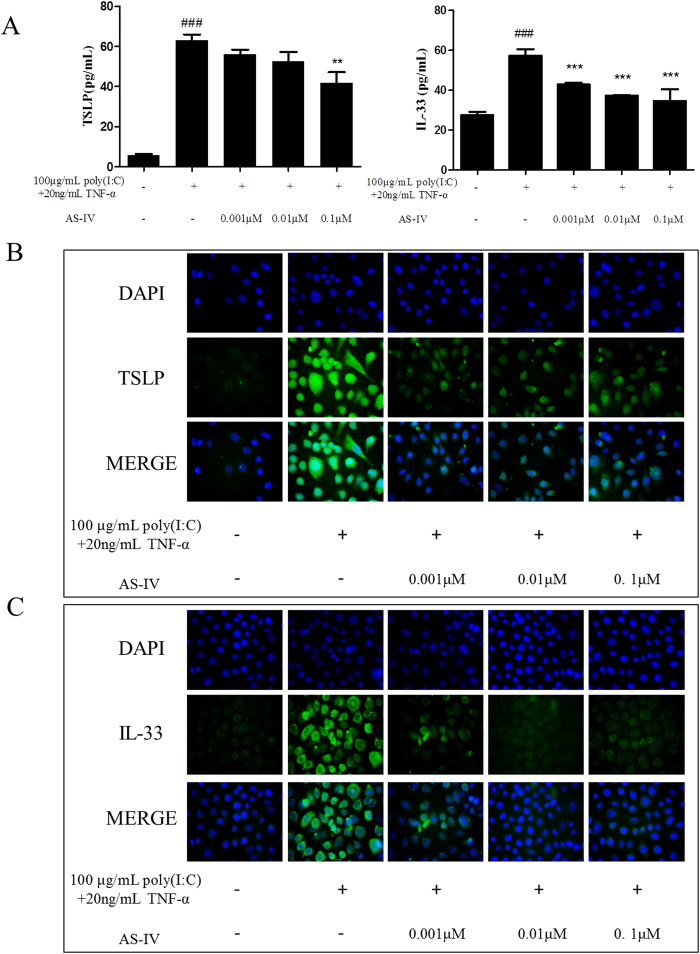
AS-IV dramatically reduced poly(I:C)- and TNF-α-induced TSLP and IL-33 production in HaCaT cells. Cells were treated with different concentrations of AS-IV and costimulated with 100 μg/mL poly(I:C) and 20 ng/mL TNF-α for 24 h. (**A**) Effects of AS-IV on protein levels of TSLP and IL-33 in HaCaT cell culture supernatants were analyzed with ELISAs (mean + SD, n = 6, ^###^P < 0.001 *vs* normal; **P < 0.01, ***P < 0.001 *vs* model). (**B**) Immunofluorescence assay was performed to confirm TSLP production in HaCaT cells (n = 5; magnification: ×400). (**C**) Immunofluorescence assay of IL-33 production (n = 5; magnification: ×400). The production of TSLP and IL-33 was inhibited by AS-IV.

HaCaT cells were treated with different concentrations of AS-IV and the costimulators poly(I:C) (100 μg/mL; Sigma, USA) and TNF-α (20 ng/mL; PeproTech) for 24 h. As shown in Fig. 7A, poly(I:C) and TNF-α significantly increased the levels of TSLP and IL-33 proteins, which were effectively downregulated by AS-IV. An immunofluorescence assay showed similar trends (Fig. 7B).

### AS-IV administered only in the initial stage markedly alleviated FITC-induced ACD occurrence and development

**Figure 8:**
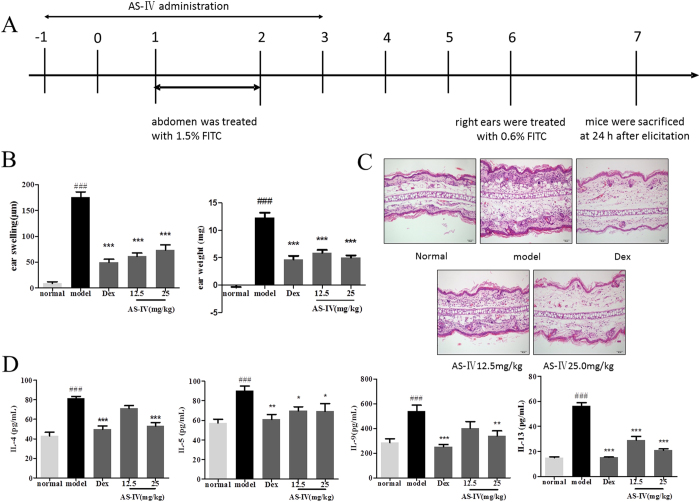
AS-IV administered only in the initial stage markedly alleviated FITC-induced ACD. (**A**) Flow charts of the FITC-induced ACD model and AS-IV was administered in the initial stage (on day −1 to day 3). The mice were killed on day 7. (**B**) Effects of AS-IV on ear swelling and ear weight in the ACD model. (**C**) Effects of AS-IV on the histopathology of H&E-stained ear skin sections (n = 5; magnification: ×200). (**D**) AS-IV given only in the initial stage reduced the levels of cytokines IL-4, IL-5, IL-9, and IL-13 in the ear tissue homogenates of the mouse model of ACD (mean + SD, n = 10, ^###^P < 0.001 *vs* normal; *P < 0.05, **P < 0.01, ***P < 0.001 *vs* model).

AS-IV downregulated the abnormally high expression of TSLP and IL-33 in the initial phase of ACD *in vivo* and inhibited these two pro-allergic cytokines in HaCaT cells *in vitro*, which we ascribed to its regulation of key pro-allergic cytokines in the initial stage of ACD, in which AS-IV inhibited ACD. To verify this, we modulated the dose regimen to observe the effects of AS-IV on the ear inflammation and Th2 cytokine expression after elicitation. AS-IV was given from day −1 to day 3 in the initial stage (Fig. 8A). By measuring ear swelling and ear weight, we demonstrated the marked alleviation of inflammation by AS-IV in mice with FITC-induced ACD (Fig. 8B). We observed very high levels of Th2 cytokines in the FITC-induced allergic ear tissues, including IL-4, IL-5, IL-9, and IL-13, and these cytokines were significantly downregulated by AS-IV (Fig. 8D). A histological analysis indicated that AS-IV administered only in the initial stage of ACD effectively alleviated FITC-induced inflammatory cell infiltration and edema (Fig. 8C).

## Discussion

Allergic diseases are among the most prevalent diseases in the 21^st^ century, with an undeniable correlation with the ever-changing environment. Therefore, it is imperative to understand the pathophysiology and treatment strategies of the many diverse allergic diseases affecting the world’s population^1^. The recurrence of allergic diseases remains the key issue for clinical treatment.

Based on the use of *A. membranaceus* to treat the recurrence of several allergic diseases, we investigated the effects of AS-IV, the major active component of *A. membranaceus*, on a model of Th2 ACD. Our results show that AS-IV inhibits the inflammation of ACD and markedly alleviates this inflammation when administered in the sensitization phase rather than in the elicitation phase, indicating that AS-IV exerts specific effects in the sensitization phase.

Because most allergic disorders are clinically observed at epidermal or mucosal surfaces, the breakdown of the physical barrier and altered innate immunity are recognized as very important in allergic reactions^29^,^30^,^31^. The epithelium plays a significant role in mucosal and skin allergies^32^ because it is the primary source of crucial cytokines and chemokines, including CCL17 (TARC), CCL22 (MDC), and IL-33, which promote Th2 cell functions, and TSLP, which interacts with DCs and mast cells to increase the Th2 response. In particular, the EC-derived cytokines IL-33 and TSLP, which are released in damaged tissues, during pathogen recognition, or after exposure to allergens, have attracted significant attention^33^. IL-33 is a nuclear cytokine of the IL-1 family, constitutively expressed in epithelial barrier tissues and lymphoid organs, which plays an important role in the type-2 innate immune response and human asthma^13^. Recent studies have demonstrated that IL-33 induces the production of large amounts of IL-5 and IL-13 by ILC2s, which initiate allergic inflammation shortly after exposure to allergens or infection by parasites or viruses^34^,^35^. IL-33 appears to function by activating ILC2s to produce large amounts of type-2 cytokines, and contributes to the occurrence and development of allergy. TSLP is highly expressed by keratinocytes in the skin lesions of patients with AD and is associated with DC activation, suggesting that TSLP is a master switch for allergic inflammation at the EC–DC interface. It also triggers DC-mediated inflammatory Th2 responses. Although the functions of IL-33 in the activation of ILC2s and the initiation of allergic inflammation in the lungs are well established, its roles in allergic and non-allergic inflammation in other tissues that express high levels of the endogenous proteins are yet to be fully explored.

We measured TSLP and IL-33 in the mouse ears on day 3 after the mice were sensitized with abdominally applied FITC. The expression of TSLP and IL-33 was significantly elevated in the ear tissues, distant from the site exposed to FITC, which is a very interesting finding. Galand *et al*.^36^ demonstrated that tape stripping induced the release of IL-33 in the skin and systemically in BALB/c mice and that the systemically released IL-33 was the primary source of the IL-33 that promoted oral anaphylaxis. A lack of Notch signaling in the mouse skin results in skin barrier defects and the significant elevation of local and serum TSLP, triggering AD and bronchial hyperresponsiveness to inhaled allergens in the absence of epicutaneous allergen sensitization^37^. The application of MC903 to the ear skin elevated the expression of TSLP not only in the mouse skin, but also in the serum. It also triggered AD and aggravated experimental allergic asthma after ovalbumin sensitization and challenge^38^. Importantly, in the present study, TSLP and IL-33 were released not only locally, but also systemically, so their levels increased in the mouse ear tissues, distant from the abdomen. However, the underlying mechanism requires further study.

Consistent with previous studies our data show that IL-33 and TSLP are highly expressed in the ACD model, especially in the initial stage, but then tend to decline in the elicitation phase compared with their expression in the initial stage of sensitization. Based on these observations, we evaluated the effects of AS-IV on the production of IL-33 and TSLP. AS-IV inhibited the expression of these key switches in the initial stage of the ACD model and reduced the proportion of ILC2s in the draining lymph nodes. Innate ILC2s in the mesenteric lymph nodes play a critical role in the generation of the adaptive Th2-cell responses to allergens^39^. ILC2-deficient mice are incapable of mounting an effective Th2-cell response. The skin is reported to contain ILC2s, which increase significantly after allergen challenge. Skin-derived ILC2s express ST2 (IL-33R) and respond to IL-33 by producing type-2 cytokines^14^. AS-IV also reduced IL-33 and TSLP expression in HaCaT cells *in vitro*, confirming its effects on these key pro-allergic cytokines.

To determine whether the effects of AS-IV on IL-33 and TSLP expression in the initial stage of ACD ultimately influence allergic inflammation, AS-IV was administered only in the initial stage. The inflammation in mice with FITC-induced ACD was markedly alleviated by AS-IV in FITC-induced ACD mice was achieved. evident as reduced ear swelling and the reduced expression of Th2 cytokines, including IL-4, IL-5, IL-9, and IL-13. A histological analysis indicated that AS-IV also effectively alleviated lymphocyte infiltration and edema. These findings suggest that the specific effects of AS-IV on IL-33 and TSLP in the initial stage of allergy affect the outcome of allergic inflammation.

In summary, our findings suggest that AS-IV effectively ameliorates allergic inflammation by regulating the expression of key pro-allergic cytokines, including IL-33 and TSLP. Given its potential utility in reducing the recurrence of allergic diseases, AS-IV could be used as a prophylactic and/or therapeutic agent for patients with a medical history of ACD, and perhaps other types of allergy. Understanding the mechanisms of IL-33 and TSLP production and their modes of action is crucial for the development of therapeutic agents that target the IL-33/ST2 and TSLP/DC pathways, with which to treat ACD and other inflammatory diseases.

## Materials and Methods

### Materials

AS-IV (purity: 99%) was purchased from Nanjing Zelang Med-Tech Co., Ltd (Nanjing, China). Dexamethasone was from TianYao Pharmaceutical Co., Ltd (Hubei, China).

### Animals

BALB/c mice were purchased from Shanghai SLAC Laboratory Animal Company (Shanghai, China). All animals were maintained at Nanjing University of Chinese Medicine under specific-pathogen-free conditions at 18–25 °C and 50–60% humidity, and were used at 6–10 weeks of age. All procedures involving animals were approved by the Animal Care and Use Committee of Nanjing University of Chinese Medicine and performed strictly according to the Guide for the Care and Use of Laboratory Animals.

### Mouse Th2-mediated ACD model

#### Whole course of the mouse Th2 mediated ACD model

After acclimatization for 3 days, the abdomens of BALB/C mice were shaved with a razor over an area of about 3 × 3 cm^2^. The abdominal skin of the mice was treated with 1.5% FITC (Sigma, St. Louis, MO, USA) in 80 μL of acetone and dibutylphthalate (1:1; vehicle) on days 1 and 2, and the right ear was treated with 20 μL of 0.6% FITC solution on day 6. On day 7 (24 h after elicitation), ear swelling was measured with a thickness gauge (7301; Mitutoyo, Kawasaki, Japan) and the mice were then killed. A patch (8 mm diameter) was punched from the left and right ears and the ear weight was calculated. The histopathological changes in the ears were examined with hematoxylin and eosin (H&E) staining. The lymph nodes draining the ear skin were isolated for flow-cytometric analysis.

#### The sensitization phase of ACD

Mice were topically sensitized with 1.5% FITC solution on the abdominal skin on days 1 and 2. Mice were treated once daily with AS-IV (6.25, 12.5, 25 and 50 mg/kg, intragastrically) and dexamethasone (0.67 mg/kg, intraperitoneally), or normal saline from day 1 to day 5.

#### The elicitation phase of ACD

As the whole course of ACD mice model, mice were sensitized and elicited by FITC, and then treated four times with AS-IV (12.5 or 25 mg/kg, intragastrically), dexamethasone (0.67 mg/kg, intraperitoneally), or normal saline at 1, 4, 8, and 23 h after elicitation on day 6.

#### The initial stage of ACD

Both the left and right ears were sensitized with 20 μL of 0.6% FITC solution on days 1 and 2. The mice were treated once daily with AS-IV (12.5 or 25 mg/kg, intragastrically), dexamethasone (0.67 mg/kg, intraperitoneally), or normal saline, from day −1 to day 3.

### Histological assessment

Ear tissue slices were fixed in 10% paraformaldehyde. Epoxy-embedded sections (4 μm) of the ear tissues were stained with H&E.

### Enzyme-linked immunosorbent assays (ELISAs) of cytokines in ear homogenates and cell culture supernatants

Both ears of each mouse were removed and ground to homogenates in ice-cold phosphate-buffered saline (PBS), and the homogenates were centrifuged at 4000 × *g* for 15 min at 4 °C. The supernatants were stored at −80 °C before analysis. The levels of IL-4, IL-5, IL-9, IL-13, TSLP, and IL-33 in the ear tissues, and those of TSLP and IL-33 in the cell culture supernatants were measured with ELISAs, according to the manufacturer’s instructions (eBioscience, San Diego, CA, USA). All measurements were made in triplicate.

### Flow-cytometric analysis of DCs and ILC2s in draining lymph nodes

The lymph nodes draining the ear skin were collected from each mouse killed 24 h after elicitation. The lymph nodes were ground with saline to prepare single-cell suspensions and the cell density was adjusted to 1–2 × 10^7^/mL. For the ILC2 analysis, the FITC anti-mouse Lineage Cocktail with Isotype Ctrl kit (BioLegend, San Diego, CA, USA) was used. The single-cell suspensions of the draining lymph nodes were stained with 20 μl fluorescently tagged antibodies against lineage markers (CD3, CD11b, CD14, CD16/CD32, and B220), 0.2 μg CD25 antibodies (eBioscience), and 0.2 μg ST2 antibodies (eBioscience), and divided into Lin^−^ and Lin^+^ populations. The cells in the Lin^−^ populations that were doubly positive for CD25 and ST2 were gated and analyzed. The proportions of Lin^−^CD25^+^ST2^+^ cells are given as the proportions of ILC2 cell. We used the isotype controls supplied with the FITC anti-mouse Lineage Cocktail with Isotype Ctrl kit (BioLegend). To quantify the activated DCs (CD11c^+^CD40^+^CD86^+^), the cells were stained with 0.2 μg of anti-CD11C antibody, 0.2 μg of anti-CD40 antibody, and 0.2 μg of anti-CD86 antibody (eBioscience) while protected from light. The cells were then washed twice with saline and resuspended in 500 μL of saline. The fluorescence-activated cell sorting analysis was performed with a FACS Accuri C6 flow cytometer (Becton, Dickinson and Company, Franklin Lakes, NJ, USA).

### Quantitative real-time PCR

The right ears were used to investigate the expression of IL-33 and TSLP mRNAs. The right ears from each group were homogenized in 1 ml of TRIzol Reagent (Life Technologies, Waltham, Massachusetts, USA) with a glass homogenizer. The total RNA was isolated according to the manufacturer’s protocol. SYBR Green PCR Master Mix (Qiagen, Hilden, Germany) was used for the real-time PCR analysis. All reactions were performed on an ABI 7500 Fast Real-Time PCR System (Applied Biosystems, Waltham, Massachusetts, USA). The oligonucleotide sequences of the PCR primers (Sangon Biotech, Shanghai, China) were 5′-TACTATACTCTCAATCCTATCCCTG-3′ (sense, S) and 5′-ACTTCTTGTGCCATTTCCTG-3′ (antisense, AS) for TSLP; 5′-TCCAACTCCAAGATTTCCCCG-3′ (S) and 5′-CATGCAGTAGACATGGCAGAA-3′ (AS) for IL-33; and 5′-GGTTGTCTCCTGCGACTTCA-3′ (S) and 5′-TGGTCCAGGGTTTCTTACTCC-3′ (AS) for glyceraldehyde-3-phosphate dehydrogenase (GAPDH). The cycle times for the genes of interest were first normalized to that of *GAPDH* in the same sample. The fold induction of gene expression was calculated with the ΔΔCT method, as previously described^40^. The results obtained for each PCR were pooled and analyzed statistically.

### Cell culture

HaCaT cells (a human keratinocyte cell line) were obtained from the Model Animal Research Center of Nanjing University (Nanjing, China). The cells were cultured in K-SFM medium (Gibco, Waltham, Massachusetts, USA) supplemented with 10% fetal bovine serum (Wisent) at 37 °C under 5% CO_2_. They were treated with different concentrations of AS-IV and costimulated with 100 μg/mL poly(I:C) and 20 ng/mL TNF-α for 24 h. The cell culture supernatants were then collected for cytokine quantification.

### Immunofluorescence assay *in vitro*

IL-33 and TSLP production was evaluated with immunofluorescence assays. HaCaT cells were starved overnight in serum-free medium, pretreated with AS-IV for 6 h or with medium (as the control), and then stimulated with 100 μg/mL poly(I:C) and 20 ng/mL TNF-α for 24 h. The cells were fixed with ice-cold paraformaldehyde for 1 h. The coverslips were washed with PBS before the cells were permeabilized with Triton X-100 (Genview, Scientific Inc., USA) and blocked with 5% bovine serum albumin for 1 h at 37 °C. The cells were probed with 4 mg/mL rabbit monoclonal antibody directed against IL-33 or TSLP (Santa Cruz Biotechnology, Santa Cruz, CA, USA) at 4 °C overnight. After repeated washes with PBS, the cells were probed with 1 mg/mL FITC-conjugated goat anti-rabbit IgG antibody (Santa Cruz Biotechnology) and 5 mg/mL 4′,6-diamidino-2-phenylindole (DAPI; Santa Cruz Biotechnology) for 1 h. The labeled sections were viewed with a fluorescence microscope (Olympus, Tokyo, Japan).

### Statistical analysis

Data are expressed as means ± standard deviations (SD). Multiple groups were compared with one-way analysis of variance and two groups with Dunnett’s test, using GraphPad Prism 5 (GraphPad Software, San Diego, CA, USA). Statistical significance was set at P < 0.05.

## Additional Information

**How to cite this article**: Bao, K.-F. *et al*. Astragaloside IV ameliorates allergic inflammation by inhibiting key initiating factors in the initial stage of sensitization. *Sci. Rep.* **6**, 38241; doi: 10.1038/srep38241 (2016).

**Publisher's note:** Springer Nature remains neutral with regard to jurisdictional claims in published maps and institutional affiliations.
